# Design of a Payload Adjustment Device for an Unpowered Lower-Limb Exoskeleton

**DOI:** 10.3390/s21124037

**Published:** 2021-06-11

**Authors:** Junghwan Yun, Ohhyun Kang, Hyun-Min Joe

**Affiliations:** 1 PCO Nhac Ltd., Kyungpook National University, Daegu 41566, Korea; junghwanyun5084@gmail.com (J.Y.); kang9822@gmail.com (O.K.); 2 Humanoid Robotics Laboratory, Department of Artificial Intelligence, Department of Robot & Smart System Engineering, Kyungpook National University, Daegu 41566, Korea

**Keywords:** human assistance, unpowered, exoskeleton, payload, variable stiffness, torque compensation

## Abstract

This paper proposes a device that can change the payload of an unpowered lower-limb exoskeleton supporting the weights of humans and loads. Our previous exoskeletons used a cam–follower structure with a spring applied to the hip joint. This exoskeleton showed satisfying performance within the payload; however, the performance decreased when the payload was exceeded. Therefore, a payload adjustment device that can adjust the wearer’s required torque by easily applying it to the cam–follower structure was developed. An exoskeleton dynamic equation that can calculate a person’s required joint torque given the required payload and the wearer’s posture was derived. This dynamic equation provides a guideline for designing a device that can adjust the allowable joint torque range of an unpowered exoskeleton. In the Adams simulation environment, the payload adjustment device is applied to the cam–follower structure to show that the payload of the exoskeleton can be changed. User convenience and mass production were taken into account in the design of this device. This payload adjustment device should flexibly change the payload of the level desired by the wearer because it can quickly change the payload of the exoskeleton.

## 1. Introduction

Exoskeletons have been studied to assist humans and have been developed for strengthening the wearer’s muscles and rehabilitation treatment [1,2,3,4,5,6,7,8]. Exoskeletons can also be classified according to their structure. First, a free-joint-type exoskeleton has a joint stiffness of 0, as measured at the joint [9,10,11,12,13]. This exoskeleton has the advantage of moving freely because there is no element hindering the wearer’s movement, but there is a limitation in that it cannot compensate for the external force acting on a person. Second, a rigid-joint-type exoskeleton has exceptionally high joint stiffness measured at the joint [14,15,16,17,18], effectively limiting joint movement. It is mainly applied to exoskeletons for rehabilitation because it can restrain the wearer’s unnecessary movements. Third, in an active-joint-type exoskeleton, actuators are applied to the joints to work in synchronization with the wearer’s intention [19,20,21,22,23,24,25,26,27]. The exoskeleton needs a battery to drive, and the actuator’s output torque must be large because the actuator must assist human movement. Finally, a quasi-passive joint type exoskeleton refers to an exoskeleton to which elements with specific stiffness are applied to the joint [28,29,30,31,32,33,34,35]. This exoskeleton has elastic elements, such as springs used to generate joint torque, and the stiffness of these elastic elements can be adjusted using an actuator.

The exoskeleton requires a large joint torque capacity to assist humans. The active-joint-type exoskeleton must apply a reducer with a high reduction ratio to the actuator to realize a large torque capacity. By using a speed reducer with a high reduction ratio, a tradeoff occurs between the torque and speed relationship and reduces the performance of the exoskeleton’s backdrivability. Therefore, the exoskeleton is prevented from responding quickly to situations where the supply power of the actuator used in the exoskeleton is cut off or a person must move rapidly [36] (e.g., a situation where the actuator does not work properly while walking, so the wearer must quickly step into a stable position or a situation where a person suddenly changes the direction of walking). This is causally related to the safety problem for the user.

Regarding an active-joint-type exoskeleton, it is known that stability and speed are indirectly proportional [37]. In other words, it can be said that the active-joint-type exoskeleton has difficulty coping with a person’s sudden movement. As a way to solve these problems to some extent, the passive-joint-type exoskeleton or the quasi-passive joint type exoskeleton can be one way to overcome this safety issue [38]. A variable stiffness actuator (VSA) has been developed to increase the allowable joint torque of a quasi-passive joint type exoskeleton [39,40,41]. The exoskeleton with a VSA can increase the torque that can be generated in the exoskeleton’s joints by controlling stiffness through an actuator. However, the quasi-passive joint type exoskeleton with a VSA requires a power source because it uses an actuator; therefore, stiffness cannot be adjusted when the power source is cut off. If the power is cut off when the stiffness is increased, the same problems as in the active-joint-type exoskeleton are caused. Additionally, using the power source requires additional components, thereby increasing the complexity of the system. Furthermore, it is not energy efficient and economical, as it consumes additional energy.

An active-joint-type exoskeleton will inevitably cause the aforementioned safety issue, is structurally complex, and has a high price [37,38]. Because actuators are also used in the quasi-passive joint type exoskeleton, problems that occur in the active-joint-type exoskeleton may also occur and increase the structural complexity due to the addition of an actuator. Considering the safety issues of the active-joint-type exoskeleton and the economy of the quasi-passive joint type exoskeleton, a passive-joint-type unpowered exoskeleton without actuators and power supplies was designed. In previous studies, it is confirmed that this designed exoskeleton has sufficient torque compensation when a person is standing or walking with an object [42]. However, in this exoskeleton system, because the payload change is not considered, it has a limitation that it cannot flexibly respond to the user’s requested payload change. Therefore, an unpowered payload adjustment device that can quickly change this payload was developed.

To design a payload adjustment device, a dynamic equation that can calculate the exoskeleton joint torque required by the wearer according to the required payload and the exoskeleton’s current posture is derived. A design guideline for fabricating an unpowered exoskeleton using this dynamic equation is proposed. In the Adams simulation environment, the validity of the dynamic equation derived was verified, and it was confirmed that the proposed payload adjustment device enables the payload adjustment.

## 2. Approach Method

### 2.1. Design Goal

**Figure 1:**
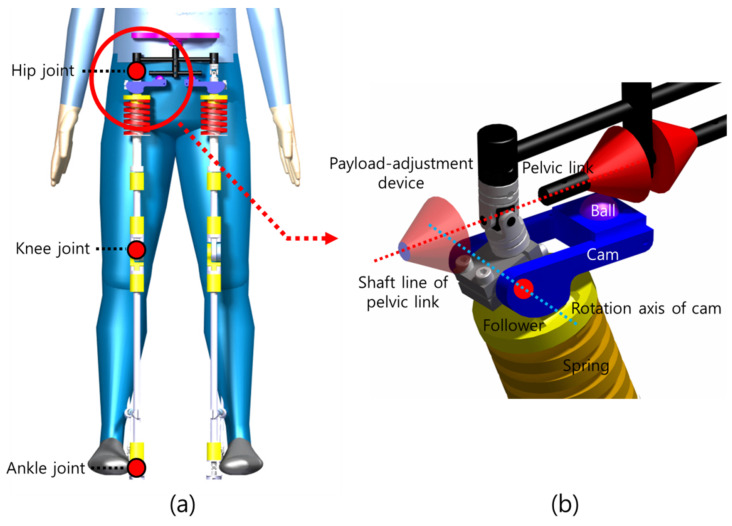
Schematic of the exoskeleton: (**a**) previous model; (**b**) new model with a payload adjustment device.

Figure 1 shows the exoskeleton designed in the previous study (Figure 1a) and the exoskeleton to which the payload adjustment device proposed in this paper is applied (Figure 1b). In Figure 1a, the exoskeleton has a hip joint, knee joint, and ankle joint and is in front of the person. Figure 1b shows the figure in which the red payload adjustment device is applied to the exoskeleton shown in Figure 1a and is an enlarged view of the hip joint shown in Figure 1a.

In Figure 1b, the exoskeleton consists of a black pelvic link, blue cam, yellow follower, spring in contact with the follower, and red payload adjustment device. The payload adjustment device can be disassembled and combined along the axis of the pelvic link. The ball mounted on the cam is in contact with the pelvic link to which the payload adjustment device is applied, and the cam rotates based on the cam’s rotation axis. This structured system creates degrees of freedom for the hip joint and enables reaction force generation through the cam–follower structure.

The only method to change the payload of the exoskeleton proposed in the previous study was to change the shape of the cam or the spring constant. Changing the spring constant has the disadvantage that it takes longer to disassemble and assemble the part, and changing the shape of the cam has the disadvantage that the structural design becomes complicated. Therefore, the design goal is set in a direction that does not increase the complexity of the design so that users can easily disassemble and assemble it.

To achieve this design goal, the reaction force generated at the contact point of the pelvic link and ball instead of changing the conventional cam shape and spring stiffness was focused on. Like the red part shown in Figure 1b, the reaction force can be changed by changing the shape of the pelvic link. The changed reaction force changes the force applied to the spring and, consequently, the payload of the exoskeleton. The red payload adjustment device in Figure 1b is combined with the shaft of the pelvic link and has no direction to the shaft line; therefore, it is easy for the exoskeleton wearer to quickly disassemble and assemble. Furthermore, this device is economical because of its uncomplicated structure and easy processing.

### 2.2. Derivation of Dynamic Equations

**Figure 2:**
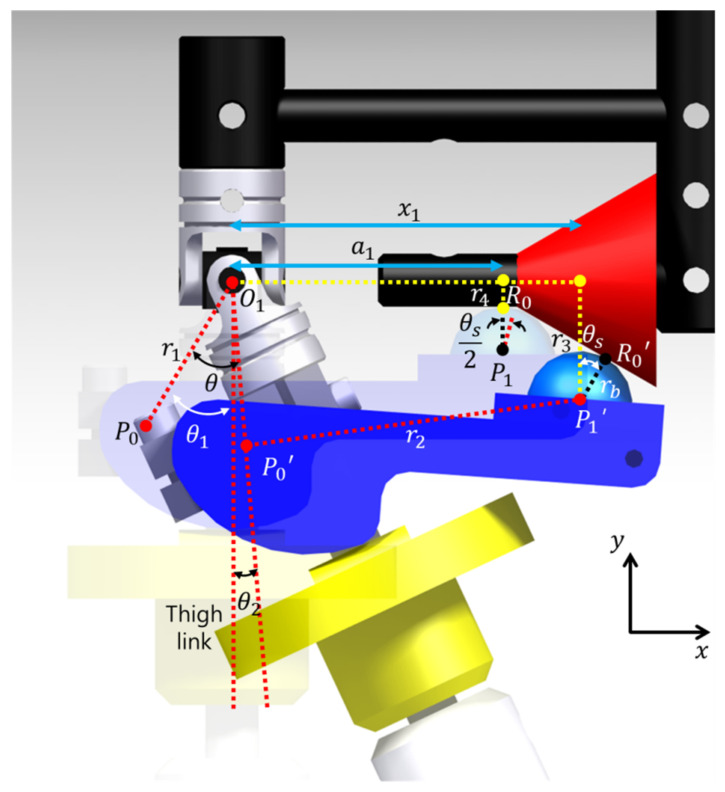
Kinematic system in the proposed exoskeleton.

To determine the shape of the payload adjustment device proposed in Section 2.1, the relationship between the shape of the payload adjustment device and the joint torque is derived. Figure 2 shows the exoskeleton to which the payload adjustment device is applied. The device is cone-shaped, and the inclination angle is set to $\theta_{s}$. The exoskeleton’s hip adduction angle is defined as θ. $O_{1}$ represents the center of rotation of the exoskeleton’s hip joint, and $P_{0}$ and $P_{0}’$ represent the cam’s center of rotation before and after hip adduction of the exoskeleton, respectively. $P_{1}$ and $P_{1}’$ represent the center of the ball before and after hip adduction of the exoskeleton, respectively, and $R_{0}$ and $R_{0}’$ represent the contact point between the ball and the pelvic link before and after hip adduction of the exoskeleton, respectively. $\theta_{1}$ represents the angle between $\bar{O_{1}P_{0}}$ and the thigh link, and $\theta_{2}$ represents $\left|\theta -\theta_{1}\right|$. To derive the dynamic equation of this system, $x_{1}$ and $r_{3}$ should first be defined. The kinematic equations are

(1) $${\left(x_{1}-r_{1}\sin\theta_{2}\right)}^{2}+{\left(r_{1}\cos\theta_{2}-r_{3}\right)}^{2}=r_{2}^{2},$$

(2) $$r_{3}-\frac{r_{b}}{\cos\theta_{s}}=\tan\theta_{s}x_{1}+r_{4}-\tan\theta_{s}\left(a_{1}+r_{b}\tan\frac{\theta_{s}}{2}\right),$$

Therefore, $x_{1}$ and $r_{3}$ are defined using Equations (1) and (2). Here $r_{1}$ represents $\bar{O_{1}P_{0}}$, and $r_{2}$ represents $\bar{P_{0}’P_{1}’}$. $r_{3}$ represents the y-axis distance from $O_{1}$ to $P_{1}’$, and $r_{4}$ represents the y-axis distance from $O_{1}$ to $R_{0}$. $r_{b}$ represents the radius of the ball. $a_{1}$ represents the x-axis distance from $O_{1}$ to $P_{1}$ when the hip adduction angle (θ) is 0 rad, and $x_{1}$ represents the x-axis distance from $O_{1}$ to $P_{1}’$ when the hip adduction angle is θ rad (θ ≠ 0 rad).

**Figure 3:**
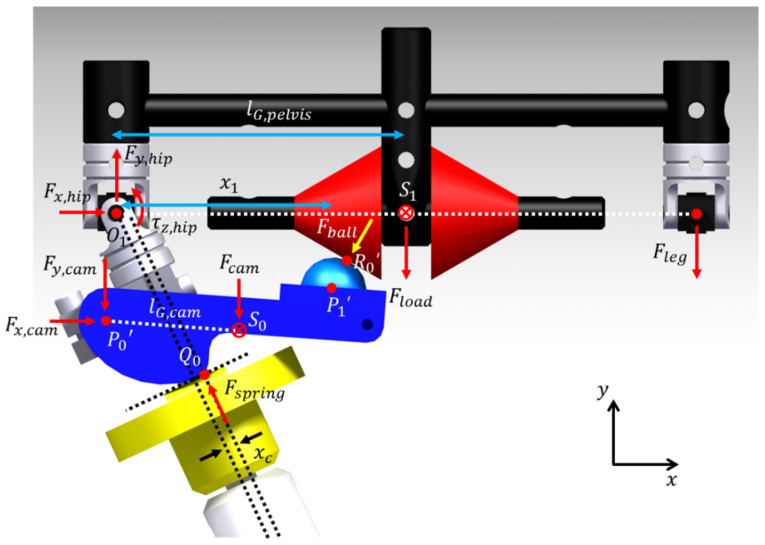
Dynamic system in the proposed exoskeleton.

Figure 3 shows the force generated at the joint and contact point when the exoskeleton’s hip adduction angle is θ. $Q_{0}$ represents the contact point of the cam and follower, and $S_{0}$ represents the cam’s center of mass. $S_{1}$ represents the pelvic link’s center of mass. $l_{G,pelvis}$ represents $\bar{O_{1}S_{1}}$, and $l_{G,cam}$ represents $\bar{P_{0}’S_{0}}$. $F_{x,hip}$ represents the x-axis reaction force generated in $O_{1}$, $F_{y,hip}$ represents the y-axis reaction force generated in $O_{1}$, and $\tau_{z,hip}$ represents the z-axis reaction torque generated in $O_{1}$. $F_{x,cam}$ represents the x-axis reaction force generated in $P_{0}’$, and $F_{y,cam}$ represents the y-axis reaction force generated in $P_{0}’$. $F_{cam}$ represents the weight of the cam acting on $S_{0}$. $F_{spring}$ represents the spring force acting on $Q_{0}$. $F_{ball}$ represents the reaction force generated at $R_{0}’$. $F_{load}$ represents the weight of an object carried by a person acting on $S_{1}$, and $F_{leg}$ represents the weight of one leg of the exoskeleton.

The single support phase that requires the most energy when a person walks is focused on, and the system shown in Figure 3 is a model assuming this situation. Compared to $F_{load}$, $F_{leg}$ is assumed to be the point mass because the force is small. The hip abduction torque ($\tau_{z,hip}$) for the hip adduction angle (θ) is defined as

(3) $$\tau_{z,hip}=F_{x,hip}\left(r_{3}-r_{b}\cos\theta_{s}\right)+F_{y,hip}\left(x_{1}+r_{b}\sin\theta_{s}\right)+F_{load}\left(l_{G,pelvis}-x_{1}-\;r_{b}\sin\theta_{s}\right)+F_{leg}\left(2l_{G,pelvis}-x_{1}-r_{b}\sin\theta_{s}\right)+I_{G,pelvis}\ddot{\theta },$$

Therefore, the hip abduction torque ($\tau_{z,hip}$) for the hip adduction angle (θ) according to the inclination angle ($\theta_{s}$) is defined using Equation (3).

### 2.3. Assembly Process of the Proposed Device

**Figure 4:**
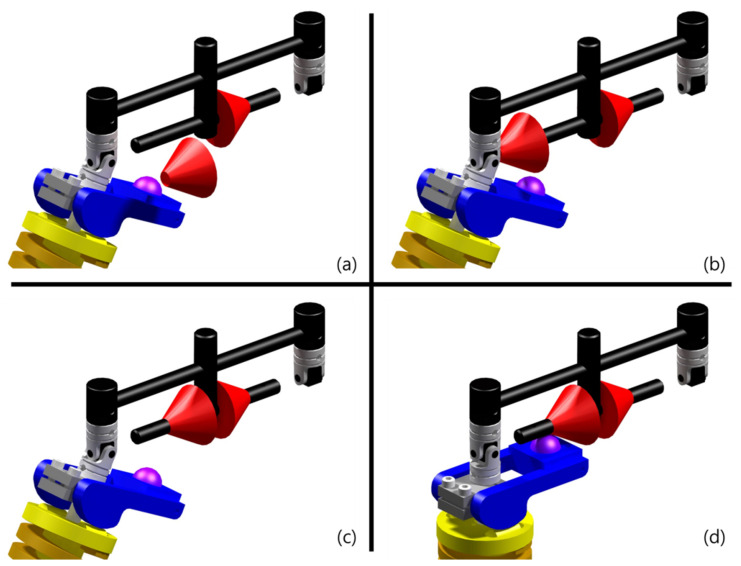
Assembly diagram of the payload adjustment device: (**a**) a step before the payload adjustment device is applied; (**b**) axial alignment of the axis of the pelvic link and the payload adjustment device; (**c**) coupling of the pelvic link and the payload adjustment device; (**d**) a step after the payload adjustment device is applied.

Figure 4a–d shows the assembly diagram of the payload adjustment device. The assembly sequence proceeds sequentially. Figure 4a shows the exoskeleton before the payload adjustment device is applied. Because there is no constraint between the ball and the pelvic link in this system, it is not difficult to perform hip abduction at the hip joint of the exoskeleton. After hip abduction of the exoskeleton is performed, there is a space between the hip joint and the pelvic link to assemble the payload adjustment device (Figure 4b). After that, coupling is performed by matching the axis of the payload adjustment device with the axis of the pelvic link (Figure 4c). Finally, when the payload adjustment device is positioned near the center of the pelvic link and the exoskeleton is positioned before hip abduction, the assembly of the payload adjustment device is completed (Figure 4d).

## 3. Simulation

**Figure 5:**
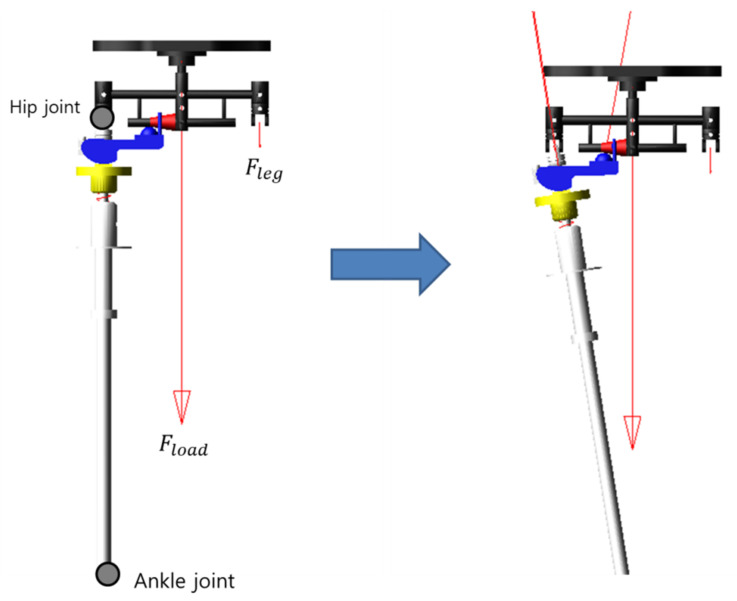
Adams simulation environment.

**Figure 6:**
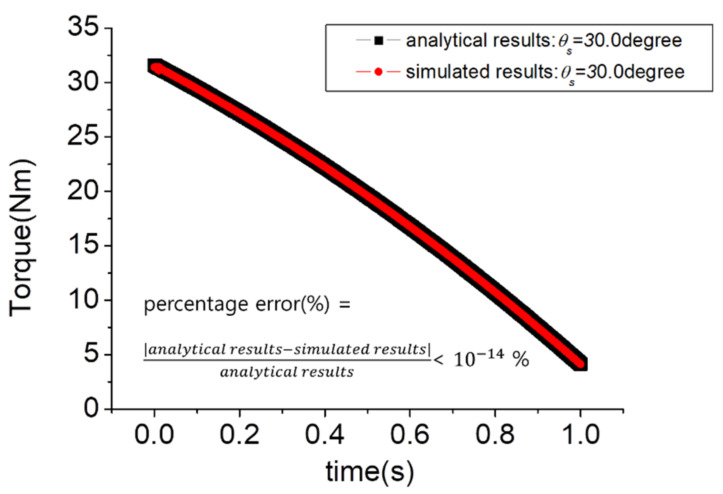
Comparison of analytical and simulated results.

A dynamics simulation in the Adams environment to prove the validity of the calculated dynamics equations was performed. Similar to Section 2.2, the experimental environment was constructed, assuming a single support phase during the human gait cycle (Figure 5). To evaluate the hip abduction torque measured in the roll direction, the movement in the pitch direction was limited, and consequently, the simulation was performed by excluding the knee joint. In the simulation environment, the spring constant was set to 551 N/mm, the angular velocity at the time of hip adduction was set to 9π/180 rad/s, and the time to perform hip adduction was set to 1 s. For the pelvic link to move horizontally to the ground, the angular velocity of the ankle joint was also set to 9π/180 rad/s, and the time was set to 1 s. $F_{load}$ was set to 300 N. Figure 6 shows the result of the derived dynamic equation and the Adams simulation result. The percentage error equation was used to check the error between the two result values, and this equation is

(4) $$\mathrm{Percentage}\;\mathrm{error}\;=\frac{\left|\mathrm{analytical}\;\mathrm{results}\;-\;\mathrm{simulated}\;\mathrm{results}\right|}{\mathrm{analytical}\;\mathrm{results}},$$

From Equation (4), the percentage error was less than $10^{-14}\%$, which is negligible. Therefore, it was verified that the dynamic equation was properly derived.

**Figure 7:**
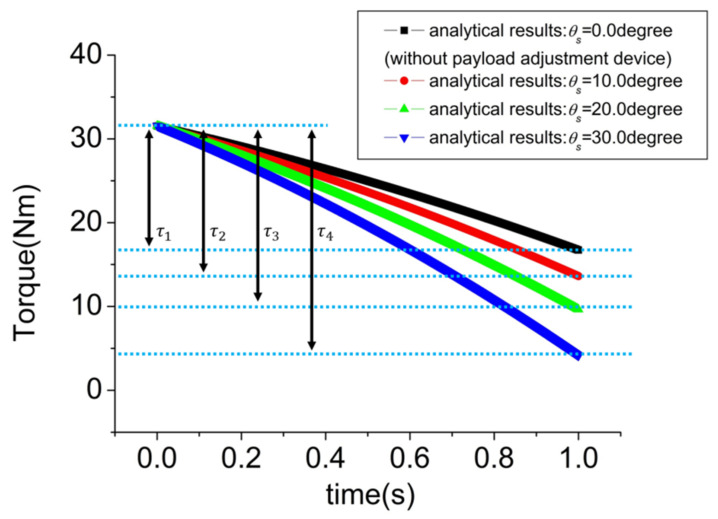
Hip joint torques when the exoskeleton with a payload adjustment device performs hip adduction.

Figure 7 is a graph showing $\tau_{z,hip}$ versus time according to $\theta_{s}$. $\tau_{i}\;\left(i=1,\;2,\;3,\;\mathrm{and}\;4\right)$ represents the difference between the maximum and minimum values of hip abduction torque according to $\theta_{s}$. Therefore, the higher this value is, the higher the payload of the exoskeleton. When $\theta_{s}$ is 0.0 degrees, it indicates the model to which the payload adjustment device is not applied. As shown in Figure 7, it was confirmed that $\tau_{i}$ increased as $\theta_{s}$ increased. Therefore, when the hip adduction angle is the same, the large $\tau_{i}$ means that the weight of the load that the spring can support is large. It was calculated as $\tau_{1}=14.8\;\mathrm{Nm}$ and $\tau_{4}=27.4\;\mathrm{Nm}$, showing a maximum torque increase rate of ~85.1%. That is, if the payload adjustment device was applied, the torque increase rate was up to 85.1%.

## 4. Discussion

Previously designed exoskeletons have shown that despite being unpowered, they can provide sufficient assistance for a person when standing or walking with objects. However, because the change of the exoskeleton’s payload was not considered in this system, there was a limit to the reduction in the exoskeleton’s performance for objects exceeding the payload. Therefore, a payload adjustment device that can flexibly change the payload of the unpowered exoskeleton was developed. Considering the safety issue when the power is cut off, this device is also designed on an unpowered basis.

Figure 4a shows the exoskeleton to which the payload adjustment device is not applied. Figure 4c shows the exoskeleton to which the payload adjustment device is applied. To increase the payload of this exoskeleton without using a payload adjustment device, it is necessary to change the cam with a different shape or change the spring constant. Both of these methods have a disadvantage in that the adaptation time is long because decomposition and coupling are complicated. However, as shown in Figure 4, the payload adjustment device proposed in this paper is considerably easier to disassemble and combine than the above-mentioned method and also has advantages in terms of cost and time for processing this device because of its simple structure. Therefore, based on these advantages, the payload adjustment device is economical and has high utility because it can quickly respond to changes in the environment.

An Adams simulation environment was constructed to verify the effectiveness of the payload adjustment device, and it was confirmed that the difference in hip abduction torque can occur up to 85.1% depending on whether the payload adjustment device is used or not. That is, it can be judged that the payload of the exoskeleton is increased. As mentioned above, the payload adjustment device proposed in this paper is easy to disassemble and assemble, so it can be replaced immediately according to the user’s usage environment. Therefore, despite the passive-joint-type exoskeleton, it can respond to various environments and can be judged to have the advantage of minimizing safety issues that occur in the active-joint-type exoskeleton.

The payload adjustment device presented in this paper has a limitation in that it cannot obtain a fully optimized torque profile because $\theta_{s}$ is constant with time. $\tau_{z,hip}$ generated in the exoskeleton to which the payload adjustment device is applied can be expressed as follows.

(5) $$\tau_{z,hip}=f\left(\theta ,\theta_{s}\right),$$

If θ and $\theta_{s}$ are assumed to be measurable variables, $\tau_{z,hip}$ generated in the exoskeleton can be calculated. In addition, if $\theta_{s}$ can be changed during hip adduction, it is expected that $\theta_{s}$ can be optimized according to the human gait cycle and the weight of the load.

## 5. Conclusions

A device that can quickly change the payload of a previously designed unpowered exoskeleton was developed. Our former exoskeleton within the payload provided sufficient assistance to a person when standing or walking with an object. However, this exoskeleton had a limit where it was difficult to change the payload. To devise a device that can flexibly change this payload, a dynamic equation from the previously designed exoskeleton with a new design variable added was derived. Experiments were conducted in an Adams simulation environment to prove the validity of the newly derived equation. Finally, the validity of the proposed kinetic equation was verified, and it was confirmed that the joint torque of the exoskeleton was controlled according to the design variable $\theta_{s}$. Therefore, it was confirmed that this could be implemented by changing $\theta_{s}$ to generate the wearer’s required level of torque. Our device can be flexibly changed according to the wearer’s working environment because disassembly and assembly can be performed quickly. Our exoskeleton, to which this device is applied, does not have an additional actuator and power supply; therefore, it is highly safe and economical. Furthermore, this device could be applied to legged robots and exoskeleton systems to control joint torque.

In future work, the proposed device is planned to be applied to the previously developed exoskeleton, and as mentioned in the discussion section, research on optimizing the shape of the proposed device according to the human gait cycle and the load is planned.

## Data Availability

Not applicable.

## Nomenclature

$x_{1}$	The x-axis distance from $O_{1}$ to $P_{1}’$
$x_{c}$	The distance from thigh link to $Q_{0}$
$r_{1}$	$\bar{O_{1}P_{0}}$ (0.03265 m)
$r_{2}$	$\bar{P_{0}’P_{1}’}$ (0.067469 m)
$r_{3}$	The y-axis distance from $O_{1}$ to $P_{1}’$
$r_{4}$	The y-axis distance from $O_{1}$ to $R_{0}$ (0.005 m)
$r_{b}$	The radius of the ball (0.005 m)
$a_{1}$	The x-axis distance from $O_{1}$ to $P_{1}$ (0.051 m)
θ	The exoskeleton’s hip adduction angle
$\theta_{s}$	The inclination angle of the payload adjustment device
$\theta_{1}$	The angle between $\bar{O_{1}P_{0}}$ and the thigh link (0.477345 rad)
$\theta_{2}$	$\left|\theta -\theta_{1}\right|$
$l_{G,pelvis}$	$\bar{O_{1}S_{1}}$ (0.0875 m)
$l_{G,cam}$	$\bar{P_{0}’S_{0}}$
$F_{x,hip}$	The x-axis reaction force generated in $O_{1}$
$F_{y,hip}$	The y-axis reaction force generated in $O_{1}$
$\tau_{z,hip}$	The z-axis reaction torque generated in $O_{1}$
$F_{x,cam}$	The x-axis reaction force generated in $P_{0}’$
$F_{y,cam}$	The y-axis reaction force generated in $P_{0}’$
$F_{cam}$	The weight of the cam acting on $S_{0}$
$F_{spring}$	The spring force acting on $Q_{0}$
$F_{ball}$	The reaction force generated at $R_{0}’$
$F_{load}$	The weight of an object carried by a person acting on $S_{1}$ (300 N)
$F_{leg}$	The weight of one leg of the exoskeleton
$I_{G,pelvis}$	Mass moment of inertia of the pelvic link ($0.002{\;\mathrm{kgm}}^{2}$)
$\ddot{\theta }$	The second derivative of θ with respect to time
$\tau_{i}$	The difference between the maximum and minimum values of hip abduction torque according to $\theta_{s}$
